# Impact of blood pressure and medication adherence on clinical outcomes in patients with hypertension

**DOI:** 10.3389/fmed.2025.1564791

**Published:** 2025-04-28

**Authors:** Hyun-Jin Kim, Byung Sik Kim, Hasung Kim, Jungkuk Lee, Jeong-Hun Shin, Ki-Chul Sung

**Affiliations:** ^1^Division of Cardiology, Department of Internal Medicine, Hanyang University College of Medicine, Hanyang University Guri Hospital, Guri, Republic of Korea; ^2^Data Science Team, Hanmi Pharm. Co., Ltd., Seoul, Republic of Korea; ^3^Division of Cardiology, Department of Internal Medicine, Kangbuk Samsung Hospital, Sungkyunkwan University School of Medicine, Seoul, Republic of Korea

**Keywords:** blood pressure, adherence, medication, hypertension, cardiovascular disease, outcome

## Abstract

**Background:**

Hypertension is a key risk factor for cardiovascular disease. Thus, effective blood pressure (BP) management and adherence to antihypertensive medications are crucial for reducing these risks in patients with hypertension. We evaluated the effect of BP and medication adherence on the clinical outcomes of patients with hypertension.

**Methods:**

This is retrospective cohort study utilized data from the Korean National Health Insurance Database. We analyzed data from 238,950 patients with hypertension aged ≥20 who underwent at least two health checkups between 2009 and 2012. Patients were categorized according to their systolic BP (SBP) and medication adherence. The primary outcome was a composite of all-cause death, myocardial infarction, ischemic stroke, hemorrhagic stroke, and hospitalization for heart failure. Cox proportional hazard models were used to estimate hazard ratios (HR) for composite outcomes.

**Results:**

Higher SBP groups were associated with increased risk of composite outcomes compared to the 120–129 mmHg group (<120 mmHg, HR 1.065; 130–139 mmHg, HR 1.056; 140–149 mmHg, HR 1.068; and ≥150 mmHg, HR 1.238). In addition, across all SBP categories, poor adherence significantly elevated the risk of composite outcomes, even after adjusting for confounding factors. Among all categories, patients with high SBP (≥150 mmHg) and poor adherence had the highest risk.

**Conclusion:**

Higher SBP and poor medication adherence were independently associated with worse clinical outcomes in patients with hypertension. Strategies to enhance medication adherence and achieve optimal BP control are essential to reduce cardiovascular risk.

## 1 Introduction

Hypertension is a major risk factor for cardiovascular diseases, including heart failure (HF), myocardial infarction (MI), and ischemic stroke—the leading causes of morbidity and mortality worldwide (1–3). These risks can be reduced by ensuring effective hypertension management, with antihypertensive medication being a cornerstone of the treatment (1). However, despite the availability of effective medications, achieving optimal blood pressure (BP) control remains challenging, with global estimates suggesting that only 50%−60% of treated hypertensive patients achieve target BP levels (4–6). One critical factor influencing BP control and subsequent clinical outcomes is medication adherence. Adherence is defined as the extent to which a patient takes medication as prescribed by their healthcare provider (7). Poor adherence to antihypertensive medication has consistently been associated with suboptimal BP control, leading to an increased risk of adverse cardiovascular events and mortality (8–10). A large cohort study using nationwide claims data demonstrated that poor adherence to antihypertensive medications was associated with significantly increased cardiovascular mortality and hospitalization risk (11). These findings highlight the strong interrelationship between adherence and BP control, reinforcing the need for strategies to improve adherence and reduce cardiovascular risk.

In clinical practice, systolic BP (SBP) levels are a primary target for hypertension management (12). Both elevated and excessively low SBP levels are associated with poor clinical outcomes, creating a complex relationship that healthcare providers must navigate (13). Therefore, understanding the interaction between SBP levels and medication adherence and how they influence clinical outcomes is essential for developing effective management strategies for patients with hypertension.

This study aims to investigate the combined impact of SBP levels and medication adherence on cardiovascular outcomes in a large, representative cohort of hypertensive patients. We hypothesize that higher SBP levels and poor adherence will independently and synergistically increase cardiovascular risk. Given the critical role of adherence in achieving BP control, this study will provide an important understanding of the need for adherence-focused interventions to optimize hypertension management and reduce cardiovascular risk.

## 2 Methods

### 2.1 Study design and population

**Figure 1:**
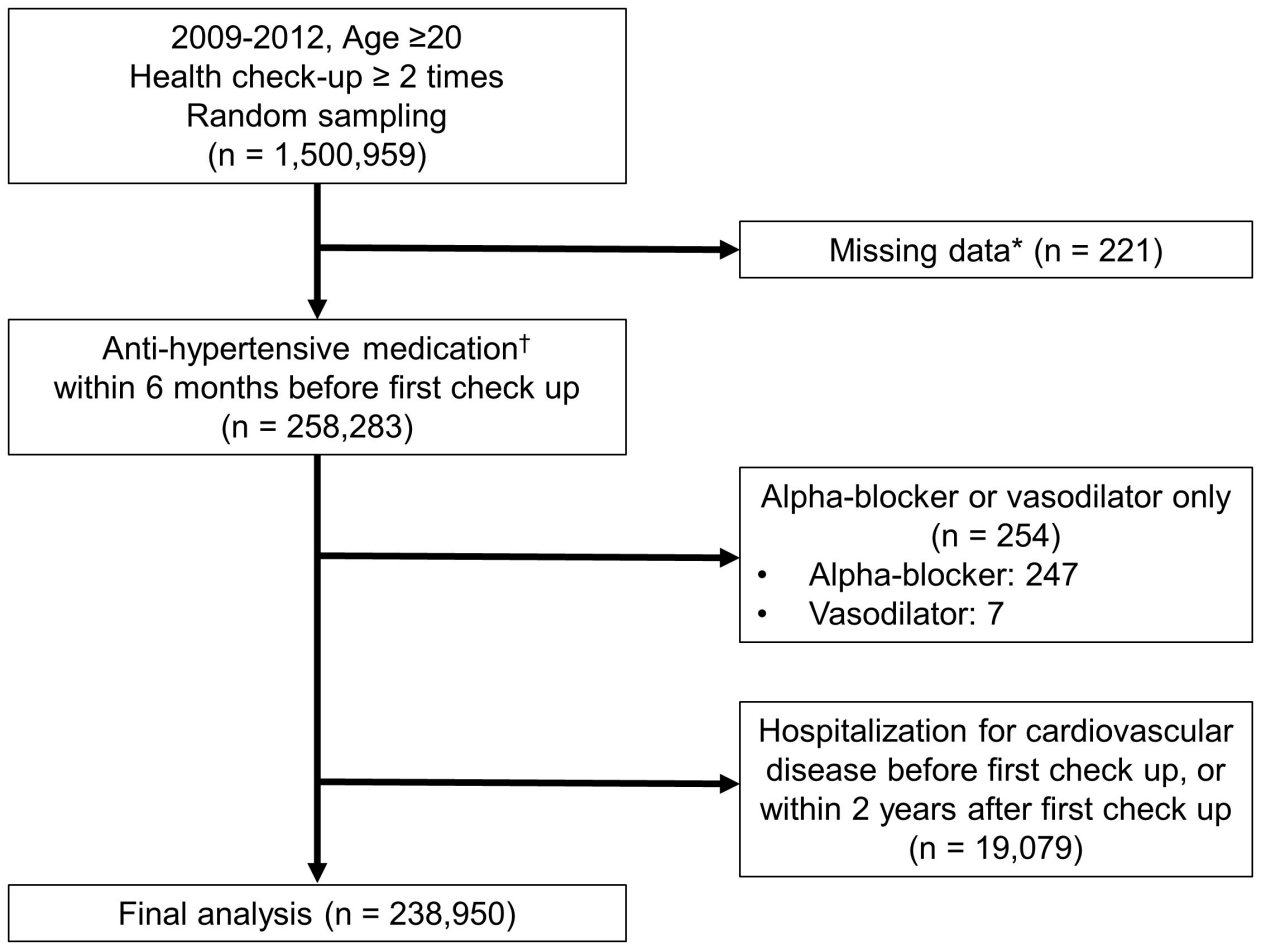
Study population. *Examined variables: systolic blood pressure (SBP), diastolic blood pressure (DBP), fasting blood glucose, cholesterol, body mass index (BMI), aspartate aminotransferase (AST), and alanine aminotransferase (ALT), gamma-glutamyl transferase (GGT), triglycerides (TG), creatinine, hemoglobin, waist circumference, and high-density lipoprotein (HDL); questionnaire items: physical activity, smoking status, and alcohol consumption. ^†^Prescription of antihypertensive medication along with diagnosis codes I10–I13, I15. Antihypertensive medications include diuretics, beta-blockers, calcium channel blockers, angiotensin-converting enzyme inhibitors (ACEi), angiotensin II receptor blockers (ARB), and others (such as alpha-blockers and vasodilators).

This retrospective cohort study utilized data from the Korean National Health Insurance Database (NHID), which includes comprehensive health information on the Korean population. The NHID is a comprehensive healthcare claims database that includes demographic information, medical diagnoses, prescriptions, procedures, and health examination records collected through the National Health Insurance Service. The NHID has been widely used for epidemiological research (14, 15). All diagnoses in the NHID were documented using the International Classification of Diseases, 10th Revision (ICD-10) codes. We focused on individuals aged ≥20 years who underwent at least two health checkups between 2009 and 2012. The individuals were identified through random sampling from the NHID. From an initial cohort of 1,500,959 individuals (Figure 1), we selected 258,283 patients who had a diagnosis of hypertension (ICD-10 codes I10–I13, I15) and had been prescribed antihypertensive medications within 6 months before their first health checkup. To ensure a well-defined study population and minimize potential bias, patients were excluded if they had missing data (*n* = 221), exclusive use of alpha-blockers or vasodilators (*n* = 254), or hospitalization for cardiovascular disease or death (*n* = 19,079) either before their first checkup or within 2 years thereafter. Alpha-blockers and vasodilators were excluded as these are not recommended as first-line antihypertensive agents and are often prescribed for specific non-hypertensive indications such as benign prostatic hyperplasia or resistant hypertension. Hospitalization for cardiovascular disease included admissions for HF (ICD-10 codes I50, I42.0, I11.0, I13.0, I13.2), MI (ICD-10 codes I21–I22), ischemic stroke (ICD-10 codes I63–I64), or hemorrhagic stroke (ICD-10 codes I60–I62), which was applied to minimize reverse causality and ensure that BP levels and medication adherence were assessed before clinical outcomes occurred. After exclusion, 238,950 patients with hypertension were included in the final study population.

The patients were stratified into five groups based on their SBP: < 120, 120–129, 130–139, 140–149, and ≥150 mmHg. Medication adherence was assessed using the medication possession ratio (MPR) and categorized as good (≥0.8), moderate (between ≥0.5 and < 0.8), or poor (< 0.5) (16). The 120–129 mmHg groups were used as the reference, representing the optimal SBP range in guidelines (1, 17). SBP was categorized in 10 mmHg increments to enable a structured analysis of BP trends and associated risks, while ≥150 mmHg was set as the highest category due to its strong association with increased cardiovascular risk and the need for intensive management. The < 120 mmHg category was included to assess potential J-curve effects, where excessively low BP may be related to adverse outcomes.

This study was approved by the local institutional review board (approval no. GURI 2024-07-008). The requirement for informed consent was waived because the NHID had obtained the participants' consent. The dataset is in the public domain and contains no individually identifiable information.

### 2.2 Data collection

The following variables were collected: age, documented as the age of the patient at the time of the first health checkup, and sex. BP measurements included both SBP and diastolic blood pressure (DBP). Smoking status was categorized as never, past, or current. Physical activity frequency was documented and categorized as 0, 1–2, 3–4, 5–6, or 7 times per week. Alcohol consumption frequency was recorded and categorized as 0, 1–2, 3–4, or ≥5 times per week. Body mass index (BMI) was categorized into < 18.5, 18.5–22.9, 23–24.9, and ≥25 kg/m^2^. Fasting glucose levels were categorized as < 100, 100–125.9, or ≥126 mg/dL, whereas the total cholesterol levels were categorized as < 200, 200–239.9, or ≥240 mg/dL. Household income was divided into quartiles, with the first and fourth quartiles representing the highest and lowest income groups.

Antihypertensive agents included diuretics, beta-blockers, calcium channel blockers, angiotensin-converting enzyme inhibitors, angiotensin II receptor blockers, and other antihypertensive agents. The number of antihypertensive agents used was categorized as 1, 2, 3, or ≥4. The use of glucose- and lipid-lowering drugs and antiplatelet agents was also documented. The Charlson comorbidity index (18), used to categorize comorbidities was calculated based on the presence and severity of 19 different medical conditions, with scores categorized into ≤ 1, 2, or ≥3. The MPR was calculated as the ratio of the number of days the medication was available to the patient to the total number of days in the observation period (16). These variables were meticulously collected and recorded to ensure a comprehensive dataset.

### 2.3 Study outcome

The primary outcome was a composite measure that included all-cause deaths, MI, ischemic stroke, hemorrhagic stroke, and hospitalization for HF during the follow-up period, which extended to December 31, 2022. The follow-up period had a mean duration of 12.35 ± 1.95 years and a median duration of 12.89 years (interquartile range: Q1 12.2 years, Q3 13.39 years). The secondary outcomes included each component of the primary composite outcome, which was analyzed individually. The causes and dates of death were obtained from the mortality records of the National Statistical Office of Korea. HF was diagnosed based on discharge diagnosis ICD-10 codes of I50, I42.0, I11.0, I13.0, and I13.2 following hospitalization, whereas MI was diagnosed in hospitalized patients who underwent coronary revascularization with discharge diagnosis ICD-10 codes of I21–I22. Ischemic stroke was diagnosed in hospitalized patients who had undergone brain imaging, with discharge diagnoses ICD-10 codes of I63 and I64. Hemorrhagic stroke was also diagnosed in hospitalized patients who had undergone brain imaging, with discharge diagnoses ICD-10 codes of I60–62.

### 2.4 Statistical analysis

Baseline patient characteristics were compared across SBP categories using appropriate statistical tests. Continuous variables are presented as mean ± standard deviation and were compared using one-way ANOVA. In contrast, categorical variables are presented as frequencies and percentages and were compared using the chi-square test.

Incidence rates were calculated as the total number of outcomes during the follow-up period per 1,000 person-years (PY). Kaplan–Meier curves were used to evaluate the impact of SBP and medication adherence on clinical outcomes, and log-rank tests were performed to compare event rates across different SBP categories and adherence levels. Hazard ratios (HRs) and 95% confidence intervals (CIs) were estimated using Cox proportional hazards regression models. SBP levels 120–129 mmHg and good medication adherence were used as reference categories. Three models were constructed to adjust for potential confounding variables: Model 1 was adjusted for age and sex; Model 2 was adjusted for age, sex, BMI, smoking status, alcohol consumption, physical activity, household income, fasting glucose, and total cholesterol; and Model 3 was adjusted for age, sex, BMI, smoking status, alcohol consumption, physical activity, household income, Charlson comorbidity index, and use of glucose- and lipid-lowering medications.

Forest plots were used to visualize HRs for the primary and secondary outcomes across the different SBP and medication adherence groups. All statistical analyses were performed using SAS version 9.4 (SAS Institute, Cary, NC), and statistical significance was set at *p*-value < 0.05.

## 3 Results

### 3.1 Baseline characteristics

Table 1 presents the baseline characteristics of the study population stratified by SBP categories. Although significant differences were observed in the variables across the SBP categories, the actual differences were relatively minor. These factors included age, sex, smoking status, physical activity, alcohol consumption, BMI, fasting glucose, total cholesterol, household income, and use of various medications. Regarding medication adherence across SBP categories, although significant differences in the adherence rates were observed (*p* < 0.0001), most patients in all SBP categories exhibited good adherence, with over 70% falling into the good adherence category. The SBP < 120, 120–129, 130–139, 140–149, and ≥150 mmHg groups had good adherence rates of 71.97%, 75.00%, 76.08%, 77.23%, and 76.30%, respectively. Hence, while statistically significant differences were observed, the clinical significance might be limited, as most patients had good medication adherence regardless of their SBP levels. Notably, a substantial number of patients in the highest SBP category (≥150 mmHg) also maintained a good medication adherence.

**Table 1:** Baseline characteristics.

**Variables**		**Systolic blood pressure (mmHg)**					***P*-value**
		<**120**	**120–129**	**130–139**	**140–149**	≥**150**	
**Total patients**	(*N* = 238,950)	44,013	54,654	77,781	32,642	29,860	
Age		59.39 ± 10.16	59.56 ± 10.26	60.26 ± 10.45	61.27 ± 10.24	62.54 ± 10.31	< 0.0001
Sex	Male	20,387 (46.32)	27,094 (49.57)	39,687 (51.02)	16,103 (49.33)	14,289 (47.85)	< 0.0001
	Female	23,626 (53.68)	27,560 (50.43)	38,094 (48.98)	16,539 (50.67)	15,571 (52.15)	
Blood pressure	SBP	111.04 ± 6.31	122.76 ± 3.18	132.94 ± 3.32	142.25 ± 2.91	157.59 ± 9.50	< 0.0001
	DBP	70.49 ± 6.92	77.07 ± 6.55	81.08 ± 6.74	85.97 ± 8.14	91.68 ± 9.96	< 0.0001
Smoking	Never	29,348 (66.68)	36,100 (66.05)	51,192 (65.82)	22,164 (67.90)	20,830 (69.76)	< 0.0001
	Past	7,437 (16.90)	9,939 (18.19)	14,571 (18.73)	6,025 (18.46)	5,113 (17.12)	
	Current	7,228 (16.42)	8,615 (15.76)	12,018 (15.45)	4,453 (13.64)	3,917 (13.12)	
Physical activity, times/week	0	27,628 (62.77)	33,695 (61.65)	48,630 (62.52)	20,806 (63.74)	19,899 (66.64)	< 0.0001
	1–2	8,435 (19.16)	10,636 (19.46)	14,714 (18.92)	5,795 (17.75)	4,805 (16.09)	
	3–4	4,545 (10.33)	5,787 (10.59)	8,006 (10.29)	3,295 (10.09)	2,709 (9.07)	
	5–6	2,103 (4.78)	2,753 (5.04)	3,862 (4.97)	1,604 (4.91)	1,413 (4.73)	
	7	1,302 (2.96)	1,783 (3.26)	2,569 (3.30)	1,142 (3.50)	1,034 (3.46)	
Alcohol consumption, times/week	0	28,657 (65.11)	34,006 (62.22)	47,266 (60.77)	20,116 (61.63)	18,763 (62.84)	< 0.0001
	1–2	10,158 (23.08)	13,287 (24.31)	18,867 (24.26)	7,359 (22.54)	6,257 (20.95)	
	3–4	3,352 (7.62)	4,792 (8.77)	7,483 (9.62)	3,240 (9.93)	2,884 (9.66)	
	≥5	1,846 (4.19)	2,569 (4.70)	4,165 (5.35)	1,927 (5.90)	1,956 (6.55)	
Body mass index, kg/m^2^	< 18.5	668 (1.52)	495 (0.91)	645 (0.83)	252 (0.77)	307 (1.03)	< 0.0001
	18.5–22.9	12,043 (27.36)	12,712 (23.26)	16,619 (21.37)	6,595 (20.20)	6,161 (20.63)	
	23–24.9	12,107 (27.51)	14,628 (26.76)	20,442 (26.28)	8,277 (25.36)	7,285 (24.40)	
	≥25	19,195 (43.61)	26,819 (49.07)	40,075 (51.52)	17,518 (53.67)	16,107 (53.94)	
Fasting glucose, mg/dL	< 100	24,377 (55.39)	29,027 (53.11)	39,500 (50.78)	15,790 (48.37)	13,683 (45.82)	< 0.0001
	100–125.9	14,199 (32.26)	18,562 (33.96)	27,364 (35.18)	11,902 (36.46)	10,922 (36.58)	
	≥126	5,437 (12.35)	7,065 (12.93)	10,917 (14.04)	4,950 (15.16)	5,255 (17.60)	
Total cholesterol, mg/dL	< 200	26,729 (60.73)	31,713 (58.03)	42,880 (55.13)	17,348 (53.15)	15,035 (50.35)	< 0.0001
	200–239.9	12,738 (28.94)	16,731 (30.61)	25,123 (32.30)	10,761 (32.97)	10,069 (33.72)	
	≥240	4,546 (10.33)	6,210 (11.36)	9,778 (12.57)	4,533 (13.89)	4,756 (15.93)	
Household income, quartiles	Q1	9,437 (21.44)	11,871 (21.72)	17,065 (21.94)	7,125 (21.83)	6,817 (22.83)	< 0.0001
	Q2	7,436 (16.90)	8,906 (16.30)	13,170 (16.93)	5,534 (16.95)	5,275 (17.67)	
	Q3	10,598 (24.08)	12,901 (23.60)	18,878 (24.27)	8,046 (24.65)	7,503 (25.13)	
	Q4	16,542 (37.58)	20,976 (38.38)	28,668 (36.86)	11,937 (36.57)	10,265 (34.38)	
Use of antihypertensive agent	Diuretics	20,720 (47.08)	25,960 (47.50)	39,641 (50.96)	17,620 (53.98)	17,961 (60.15)	< 0.0001
	Beta blockers	23,213 (52.74)	28,980 (53.02)	42,791 (55.01)	18,903 (57.91)	18,984 (63.58)	< 0.0001
	Calcium channel blockers	31,943 (72.58)	42,139 (77.10)	62,627 (80.52)	27,053 (82.88)	25,561 (85.60)	< 0.0001
	ACE inhibitors	13,436 (30.53)	16,669 (30.50)	24,986 (32.12)	11,082 (33.95)	11,232 (37.62)	< 0.0001
	Angiotensin II receptor blockers	30,679 (69.70)	38,676 (70.77)	55,402 (71.23)	24,052 (73.68)	22,888 (76.65)	< 0.0001
	Others	2,557 (5.81)	3,271 (5.98)	5,056 (6.50)	2,383 (7.30)	2,488 (8.33)	< 0.0001
Number of antihypertensive agents	1	8,117 (18.44)	9,049 (16.56)	11,102 (14.27)	3,897 (11.94)	2,560 (8.57)	< 0.0001
	2	11,447 (26.01)	14,171 (25.93)	19,025 (24.46)	7,469 (22.88)	5,738 (19.22)	
	3	11,300 (25.67)	14,244 (26.06)	20,598 (26.48)	8,516 (26.09)	7,755 (25.97)	
	≥4	13,149 (29.88)	17,190 (31.45)	27,056 (34.78)	12,760 (39.09)	13,807 (46.24)	
Use of glucose-lowering drugs		9,136 (20.76)	10,883 (19.91)	15,801 (20.31)	6,784 (20.78)	6,887 (23.06)	< 0.0001
Use of lipid-lowering drugs		19,600 (44.53)	24,183 (44.25)	33,408 (42.95)	13,987 (42.85)	12,619 (42.26)	< 0.0001
Use of antiplatelet agent		26,212 (59.56)	32,612 (59.67)	46,438 (59.70)	19,717 (60.40)	18,542 (62.10)	< 0.0001
Charlson comorbidity index	≤ 1	23,103 (52.49)	29,847 (54.61)	42,975 (55.25)	17,793 (54.51)	16,278 (54.51)	< 0.0001
	2	9,578 (21.76)	11,631 (21.28)	16,373 (21.05)	7,015 (21.49)	6,316 (21.15)	
	≥3	11,332 (25.75)	13,176 (24.11)	18,433 (23.70)	7,834 (24.00)	7,266 (24.33)	
Medication possession ratio		0.79 ± 0.26	0.82 ± 0.23	0.82 ± 0.23	0.83 ± 0.22	0.83 ± 0.22	< 0.0001
Medication adherence	Good	31,674 (71.97)	40,990 (75.00)	59,172 (76.08)	25,211 (77.23)	22,783 (76.30)	< 0.0001
	Moderate	6,558 (14.90)	8,037 (14.71)	11,019 (14.17)	4,580 (14.03)	4,447 (14.89)	
	Poor	5,781 (13.13)	5,627 (10.30)	7,590 (9.76)	2,851 (8.73)	2,630 (8.81)	

ACE, angiotensin-converting enzyme; DBP, diastolic blood pressure; SBP, systolic blood pressure.

### 3.2 Clinical outcomes according to blood pressure and medication adherence

Table 2 shows the incidence rates of the composite primary outcome per 1,000 PY across the different SBP categories and medication adherence levels. The incidence rate increased from 18.90 per 1,000 PY in the < 120 mmHg category to 26.86 per 1,000 PY in the ≥150 mmHg category. Similarly, the incidence rate increased from 20.11 per 1,000 PY in the good adherence group to 21.71 per 1,000 PY in the poor adherence group. Using SBP 120–129 mmHg as the reference category, both lower and higher SBP were associated with an increased risk for the composite outcome. Specifically, patients with SBP < 120 mmHg were at higher risk than those in the reference group (HR 1.065, 95% CI 1.037–1.094, *p* < 0.0001), and the risk progressively increased with higher SBP levels (≥150 mmHg, HR 1.238, 95% CI 1.204–1.272, *p* < 0.0001). We also found that medication adherence significantly affected clinical outcomes. Using good adherence as the reference, moderate adherence was associated with an increased risk, and poor adherence was associated with an even higher risk. Regardless of the adjusted model, higher SBP and poorer medication adherence were associated with an increased risk of the composite primary outcome.

**Table 2:** Risk of primary outcomes according to systolic blood pressure and medication adherence.

**Variables**	***n***	**Events**	**Incidence rate per 1,000 PY**	**Model 1**			**Model 2**			**Model 3**		
				**HR**	**95% CI**	***P*** **-value**	**HR**	**95% CI**	***P*** **-value**	**HR**	**95% CI**	***P*** **-value**
**SBP (mmHg)**												
< 120	44,013	9,848	18.90	1.089	1.060–1.118	< 0.0001	1.077	1.048–1.106	< 0.0001	1.065	1.037–1.094	< 0.0001
120–129	54,654	11,697	18.01	1 (Ref.)	–		1 (Ref.)	–		1 (Ref.)	–	
130–139	77,781	18,453	20.10	1.051	1.027–1.076	< 0.0001	1.050	1.026–1.075	< 0.0001	1.056	1.031–1.080	< 0.0001
140–149	32,642	8,203	21.49	1.058	1.029–1.089	< 0.0001	1.060	1.030–1.090	< 0.0001	1.068	1.038–1.099	< 0.0001
≥150	29,860	9,147	26.86	1.231	1.198–1.265	< 0.0001	1.219	1.185–1.253	< 0.0001	1.238	1.204–1.272	< 0.0001
**Adherence**												
Good	179,830	42,636	20.11	1 (Ref.)	–		1 (Ref.)	–		1 (Ref.)	–	
Moderate	34,641	8,523	21.02	1.196	1.168–1.224	< 0.0001	1.196	1.168–1.224	< 0.0001	1.201	1.173–1.229	< 0.0001
Poor	24,479	6,189	21.71	1.266	1.232–1.300	< 0.0001	1.269	1.235–1.303	< 0.0001	1.262	1.229–1.297	< 0.0001
Interaction	SBP x	Adherence				0.9600			0.9738			0.9447

CI, confidence interval; HR, hazard ratio; SBP, systolic blood pressure; PY, person-year.

Model 1: adjusted for age and sex.

Model 2: adjusted for age, sex, body mass index, smoking status, alcohol consumption, physical activity, household income, fasting glucose, and total cholesterol.

Model 3: adjusted for age, sex, body mass index, smoking status, alcohol consumption, physical activity, household income, Charlson comorbidity index, and use of glucose-and lipid-lowering drugs.

### 3.3 Interaction between SBP and adherence on risk of clinical outcome

Table 3 shows the interaction between SBP and adherence within each SBP category, with good adherence as the reference group. Across all SBP categories, poor medication adherence was associated with a higher risk of adverse clinical outcomes. In Model 3, patients in the SBP < 120 mmHg category who had poor adherence exhibited a significantly higher risk than those with good adherence. Similarly, in the SBP 120–129 mmHg category, poor adherence was associated with an increased risk, and this trend persisted in higher SBP categories.

**Table 3:** Risk of primary outcomes stratified by systolic blood pressure and medication adherence.

**SBP (mmHg)**	**Adherence**	***n***	**Events**	**Incidence rate per 1,000 PY**	**Model 1**			**Model 2**			**Model 3**		

					**HR**	**95% CI**	***P*** **-value**	**HR**	**95% CI**	***P*** **-value**	**HR**	**95% CI**	***P*** **-value**
< 120	Good	31,674	6,964	18.51	1 (Ref.)	–		1 (Ref.)	–		1 (Ref.)	–	
	Moderate	6,558	1,518	19.66	1.175	1.111–1.242	< 0.0001	1.18	1.117–1.248	< 0.0001	1.184	1.12–1.251	< 0.0001
	Poor	5,781	1,366	20.18	1.262	1.191–1.337	< 0.0001	1.272	1.200–1.348	< 0.0001	1.249	1.178–1.325	< 0.0001
120–129	Good	40,990	8,635	17.68	1 (Ref.)	–		1 (Ref.)	–		1 (Ref.)	–	
	Moderate	8,037	1,737	18.28	1.193	1.133–1.256	< 0.0001	1.196	1.136–1.259	< 0.0001	1.194	1.134–1.258	< 0.0001
	Poor	5,627	1,325	20.08	1.293	1.22–1.37	< 0.0001	1.304	1.23–1.382	< 0.0001	1.285	1.212–1.362	< 0.0001
130–139	Good	59,172	13,888	19.85	1 (Ref.)	–		1 (Ref.)	–		1 (Ref.)	–	
	Moderate	11,019	2,691	20.80	1.192	1.143–1.242	< 0.0001	1.189	1.14–1.239	< 0.0001	1.194	1.145–1.244	< 0.0001
	Poor	7,590	1,874	21.13	1.254	1.195–1.316	< 0.0001	1.251	1.192–1.313	< 0.0001	1.249	1.19–1.311	< 0.0001
140–149	Good	25,211	6,268	21.22	1 (Ref.)	–		1 (Ref.)	–		1 (Ref.)	–	
	Moderate	4,580	1,163	21.84	1.19	1.118–1.267	< 0.0001	1.193	1.12–1.27	< 0.0001	1.205	1.131–1.283	< 0.0001
	Poor	2,851	772	23.37	1.255	1.165–1.352	< 0.0001	1.252	1.162–1.35	< 0.0001	1.264	1.173–1.363	< 0.0001
≥150	Good	22,783	6,881	26.43	1 (Ref.)	–		1 (Ref.)	–		1 (Ref.)	–	
	Moderate	4,447	1,414	27.99	1.23	1.162–1.303	< 0.0001	1.224	1.155–1.296	< 0.0001	1.237	1.168–1.311	< 0.0001
	Poor	2,630	852	28.76	1.287	1.199–1.382	< 0.0001	1.277	1.189–1.372	< 0.0001	1.302	1.212–1.399	< 0.0001

CI, confidence interval; HR, hazard ratio; SBP, systolic blood pressure; PY, person-year.

Model 1: adjusted for age and sex.

Model 2: adjusted for age, sex, body mass index, smoking status, alcohol consumption, physical activity, household income, fasting glucose, and total cholesterol.

Model 3: adjusted for age, sex, body mass index, smoking status, alcohol consumption, physical activity, household income, Charlson comorbidity index, and use of glucose-and lipid-lowering drugs.

**Figure 2:**
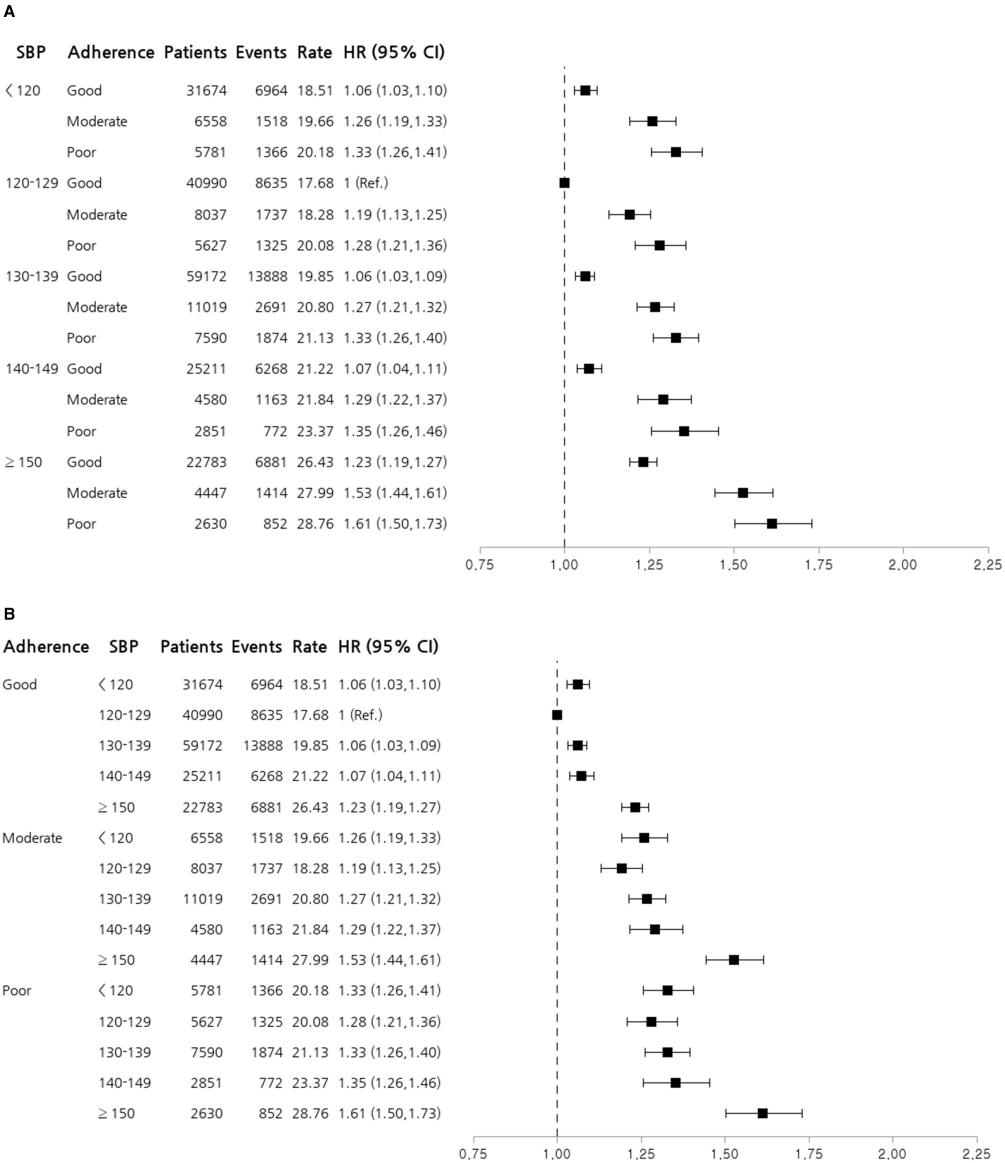
Forest plot stratified by systolic blood pressure and medication adherence. **(A)** Forest plot of HRs for the composite primary outcome according to SBP categories and medication adherence levels. **(B)** Forest plot of HRs for the composite primary outcome according to medication adherence levels and SBP categories. HRs shown are adjusted using Model 3, including age, sex, body mass index, smoking status, alcohol consumption, physical activity, household income, Charlson comorbidity index, and use of glucose- and lipid-lowering drugs. The reference group had an SBP of 120–129 mmHg and good adherence. The plot illustrates the increased risk associated with higher SBP and poorer adherence to antihypertensive medication. CI, confidence interval; HR, hazard ratio; SBP, systolic blood pressure.

Figure 2A illustrates HRs for the composite primary outcome according to SBP categories and medication adherence levels, adjusted using Model 3. Using SBP 120–129 mmHg with good adherence as the reference group, the highest risk was observed in patients with SBP ≥150 mmHg and poor adherence (HR 1.61, 95% CI 1.50–1.73). Both low and very high SBP were associated with an increased risk, with the most pronounced risk in the high SBP (≥150mmHg) and poor adherence groups. Figure 2B shows HRs for the composite primary outcomes according to medication adherence levels and SBP categories. A trend in which the risk increases with poorer adherence and higher SBP was observed. Interestingly, minimal differences in HRs were observed among patients with good medication adherence across various SBP categories. Therefore, maintaining good medication adherence is crucial for minimizing adverse outcomes, especially in patients with high SBP.

The secondary outcomes were the following individual components of the primary composite outcome: all-cause death, MI, ischemic stroke, hemorrhagic stroke, and hospitalization for HF. Supplementary Figures S1–S5 consistently show that poor adherence to antihypertensive medication and higher SBP were associated with an increased risk of these adverse outcomes. Notably, in patients with SBP 120–129 mmHg and good adherence showed statistically significant favorable outcomes in all-cause death, ischemic stroke, and HF hospitalization compared to other BP or adherence groups. These findings suggest the importance of maintaining optimal BP and good medication adherence to minimize mortality risk and major cardiovascular events.

## 4 Discussion

In this study, we used data from the Korean NHID to investigate the impact of SBP and medication adherence on the clinical outcomes of patients with hypertension. Our findings indicate that both higher SBP and poorer medication adherence significantly increase the risk of adverse clinical outcomes, including all-cause deaths, MI, ischemic stroke, hemorrhagic stroke, and hospitalization for HF. The effect of medication adherence is particularly essential, as patients with good medication adherence showed minimal increase in the risk of adverse clinical outcomes across different SBP levels. Conversely, patients with poor adherence exhibited a significantly increased risk, particularly in the group with poor BP control. Patients with SBP ≥150 mmHg and poor adherence exhibited the highest risk of adverse clinical outcomes. Taken together, our findings suggest the importance of maintaining optimal BP control and good medication adherence to mitigate the risk of mortality and major cardiovascular events.

Patients with SBP 120–129 mmHg generally had the lowest risk of adverse outcomes, whereas those with lower (< 120 mmHg) and higher (≥150 mmHg) SBP had increased risks. This risk was significantly higher in patients with poor medication adherence across all SBP categories. Our results align with the American Heart Association's scientific statement, which emphasizes the essential role of medication adherence in managing hypertension and achieving BP control (19). The American Heart Association's scientific statement also highlights that non-adherence significantly contributes to poor BP control and increased cardiovascular risk. Recent studies corroborate these findings, demonstrating that patients who adhere to their antihypertensive medication regimens have better BP control and lower rates of cardiovascular events and mortality (4). A meta-analysis by Naderi et al. (9) reported that improved adherence was associated with a substantial reduction in the risk of cardiovascular events, further highlighting the importance of medication adherence.

Notably, most patients (>70%) across all SBP categories in our study exhibited good medication adherence. This may explain why the outcome differences between certain SBP groups, such as the 140–149 and 130–139 mmHg groups, were not as pronounced as expected. The white-coat uncontrolled hypertension (WUCH) phenomenon, where patients demonstrated higher BP readings in clinical settings but maintained better control outside of these environments, might have also influenced these results (20). Similarly, for patients with SBP < 120 mmHg, WUCH might lead to an overestimation of risk, suggesting the possibility of a lower actual risk than that observed. As our study population included patients already receiving antihypertensive medication, these findings may also reflect differences in BP management across the SBP categories. Future studies using continuous BP monitoring, such as wearable devices, could provide more precise assessments and clarify these trends.

Böhm et al. (13) illustrated a J-curve phenomenon in which excessively low and high SBP levels were correlated with adverse outcomes. Similarly, our findings indicated that patients with SBP < 120 mmHg and SBP ≥150 mmHg had an increased risk of adverse clinical outcomes, although the risk increase at lower SBP (< 120 mmHg) was not as pronounced as that at higher SBP (≥150 mmHg). This J-curve relationship has been consistently reported in multiple studies and guidelines, emphasizing that excessively low SBP may be associated with increased cardiovascular risk, particularly in patients with established cardiovascular disease. Furthermore, the 2017 American College of Cardiology (ACC)/American Heart Association (AHA) hypertension guideline (1), 2023 European Society of Hypertension (ESH) guideline (17), and 2024 European Society of Cardiology (ESC) guideline (21) recommend a target SBP < 130 mmHg but emphasize the need for individualized treatment, particularly in older or frail patients, to avoid hypoperfusion-related complications at very low SBP levels. One possible explanation for this phenomenon is that excessively low SBP may lead to inadequate organ perfusion, particularly affecting the coronary and cerebral circulation, increasing the risk of myocardial ischemia and stroke (22–25). In our study, we observed that while SBP < 120 mmHg was associated with higher risk, this risk was mitigated in patients with good medication adherence, suggesting that the adverse outcomes in this group might be partially influenced by poor adherence and uncontrolled comorbid conditions rather than BP alone. To further clarify this relationship, we subdivided the < 120 mmHg group into < 110 mmHg and 110–119 mmHg. The < 110 mmHg group exhibited a higher risk (HR 1.178), while the 110–119 mmHg group had significantly lower risk and even a lower HR than the 130–139 mmHg group (HR 1.034 vs. 1.056, respectively; Supplementary Table S1). This trend was most clearly observed in the good adherence group, where a distinct J-curve pattern was evident—patients with SBP < 110 mmHg had a higher risk (HR 1.191), while those with SBP 110–119 mmHg had lower risk than the 130–139 mmHg group (Supplementary Table S2). However, in the moderate and poor adherence groups, the J-curve pattern was less pronounced, with statistically significant differences primarily seen at the extremes of SBP (< 110 mmHg and ≥150 mmHg), suggesting that poor adherence may attenuate or confuse the expected SBP-outcome relationship. Hence, for most patients, the primary focus remains avoiding high BP rather than targeting excessively low BP levels, especially because clinical outcome benefits mainly result from maintaining good medication adherence. The interplay between SBP and medication adherence emphasizes the need for tailored hypertension management strategies. Our findings show that poor adherence, regardless of the SBP category, consistently leads to a higher risk of adverse outcomes. For instance, in the SBP < 120 mm Hg category, patients with good adherence had better outcomes than those with poor adherence, emphasizing the protective effect of consistent good medication adherence. Addressing optimal SBP targets and enhancing medication adherence are crucial for mitigating risks and improving prognosis in patients with hypertension (26).

Several studies have highlighted the critical role of medication adherence in determining the clinical outcomes of patients with hypertension. Tajeu et al. (27) demonstrated that lower rates of antihypertensive medication persistence and adherence among adults aged < 65 years were associated with a significantly higher risk of cardiovascular events. They also highlighted that promoting medications associated with better adherence and using extended prescription periods could help improve persistence and adherence rates. Similarly, Kim et al. (11) reported that poor adherence (< 50%) to antihypertensive medication was associated with a markedly higher risk of cardiovascular mortality and hospitalization in the Korean population. This study showed that even intermediate adherence levels (50% ≤ to < 80%) significantly increased the risk of adverse outcomes compared to good adherence (≥80%), emphasizing the importance of maintaining consistent medication intake. Moreover, Kronish et al. (28) also found that non-adherence was associated with greater BP variability between clinic visits and independently associated with increased cardiovascular risk. This BP variability caused by non-adherence emphasizes the importance of consistent medication use to achieve stable BP control and prevent adverse cardiovascular events. Given the significant impact of adherence on clinical outcomes, several targeted interventions can help improve medication adherence in hypertensive patients. Simplifying drug regimens through fixed-dose combination therapy reduces pill burden and improves adherence, as demonstrated in multiple studies (29–31). Digital health tools, including mobile apps and telemonitoring systems, can facilitate adherence tracking, provide reminders, and enhance patient engagement (32). Additionally, behavioral strategies such as motivational interviewing and pharmacist-led counseling have been shown to improve adherence by addressing psychological and practical barriers (33–35). Taken together, non-adherence to antihypertensive medications is a significant modifiable risk factor for poor clinical outcomes, including all-cause death, MI, stroke, and HF. Our findings confirmed the importance of adherence by demonstrating that patients with poor adherence, particularly those with high SBP (≥150 mmHg), had the highest risk of adverse outcomes. Implementing these adherence-improving strategies in clinical practice may help optimize BP control and reduce cardiovascular risk in hypertensive patients.

Despite the significant findings of this study, there are several limitations worth noting. First, the retrospective study design inherently carries risks of bias and confounding factors. Second, medication adherence was assessed using MPR, which, while commonly used, does not fully capture actual medication intake or reasons for non-adherence (36). MPR reflects medication availability but does not measure whether patients take their prescriptions correctly. Alternative measures, such as the proportion of days covered, are often preferred for adherence assessment as they consider continuous coverage over time. Additionally, patient-reported adherence surveys can provide further insights into behavioral aspects of adherence, such as intentional non-adherence. Furthermore, while MPR has been widely validated in previous epidemiological studies, it was not independently revalidated within this study. Given the retrospective design and claims-based dataset, we applied standard MPR methodology without conducting a separate validation process. Future research should incorporate multiple adherence measures for a more comprehensive evaluation. Third, the study population comprised individuals who participated in regular health checkups. This may introduce selection bias, as such individuals might be more health-conscious and adhere to medical advice than the general hypertensive population. Fourth, differences in healthcare systems, medication accessibility, and adherence behaviors may influence the generalizability of our results. Korea's universal healthcare system ensures broad access to antihypertensive medications, potentially leading to higher adherence rates than countries with privatized or fragmented systems, where financial barriers may impact adherence. Genetic, dietary, and lifestyle factors may also contribute to BP variability across populations. Future research should compare these findings with multi-ethnic cohorts and diverse healthcare settings to confirm their global applicability. Finally, residual confounding may remain despite the comprehensively adjusted statistical models, as unmeasured variables such as dietary habits, stress levels, and other lifestyle factors could influence BP control and clinical outcomes.

Nevertheless, our study has several strengths that mitigate these limitations. We included patients who had started antihypertensive medication within 6 months before their first health checkup, which effectively reduced bias related to the long-term effects of hypertension or extended medication use. Our findings more accurately reflect current BP control and adherence patterns by selecting a population early in the hypertension management journey. Additionally, we used a large nationwide dataset from The Korean NHID, which enhanced the robustness and generalizability of our results to a broader population, providing valuable insights for hypertension management strategies.

## 5 Conclusion

In conclusion, although maintaining SBP within the optimal range is important, we highlighted the critical role of medication adherence in reducing the risk of death and major cardiovascular events in patients with hypertension. Poor medication adherence was associated with worse outcomes across all SBP categories, emphasizing the need for comprehensive management strategies that prioritize both BP control and sustained adherence. Key interventions include fixed-dose combination therapy to simplify regimens, digital health tools for adherence tracking, and behavioral strategies such as pharmacist-led counseling. A multidisciplinary care model incorporating regular follow-ups and patient education can further optimize hypertension management. Our findings emphasize that targeting both BP control and adherence is essential for reducing adverse cardiovascular outcomes. Future research should explore effective adherence-enhancing strategies in various healthcare settings.

## Data Availability

The datasets analyzed for this study can be found, upon reasonable request, from the corresponding author. Requests to access these datasets should be directed to Jeong-Hun Shin, cardio.hyapex@gmail.com.

## References

[1] Whelton PK Carey RM Aronow WS Casey DE Jr. Collins KJ Dennison Himmelfarb C. 2017 ACC/AHA/AAPA/ABC/ACPM/AGS/APhA/ASH/ASPC/NMA/PCNA guideline for the prevention, detection, evaluation, and management of high blood pressure in adults: a report of the American college of cardiology/American heart association task force on clinical practice guidelines. Hypertension. (2018) 71:e13–115. 10.1161/HYP.0000000000000065 29133356

[2] Collaborators GBDRF. Global burden of 87 risk factors in 204 countries and territories, 1990-2019: a systematic analysis for the global burden of disease study 2019. Lancet. (2020) 396:1223–49. 10.1016/S0140-6736(20)30752-2 33069327 PMC7566194

[3] Kim HC Lee H Lee HH Son D Cho M Shin S. Korea hypertension fact sheet 2023: analysis of nationwide population-based data with a particular focus on hypertension in special populations. Clin Hypertens. (2024) 30:7. 10.1186/s40885-024-00262-z 38424634 PMC10905929

[4] Burnier M Egan BM. Adherence in Hypertension. Circ Res. (2019) 124:1124–40. 10.1161/CIRCRESAHA.118.313220 30920917

[5] Kario K Okura A Hoshide S Mogi M. The WHO Global report 2023 on hypertension warning the emerging hypertension burden in globe and its treatment strategy. Hypertens Res. (2024) 47:1099–102. 10.1038/s41440-024-01622-w 38443614

[6] Kim HC Lee H Lee HH Ahn SV Lee JM Cheon DY. Korea hypertension fact sheet 2024: nationwide population-based analysis with a focus on young adults. Clin Hypertens. (2025) 31:e11. 10.5646/ch.2025.31.e11 40083596 PMC11903208

[7] Jimmy B Jose J. Patient medication adherence: measures in daily practice. Oman Med J. (2011) 26:155–9. 10.5001/omj.2011.38 22043406 PMC3191684

[8] Nieuwlaat R Wilczynski N Navarro T Hobson N Jeffery R Keepanasseril A. Interventions for enhancing medication adherence. Cochrane Database Syst Rev. (2014) 2014:CD000011. 10.1002/14651858.CD000011.pub4 25412402 PMC7263418

[9] Naderi SH Bestwick JP Wald DS. Adherence to drugs that prevent cardiovascular disease: meta-analysis on 376,162 patients. Am J Med. (2012) 125:882–7e1. 10.1016/j.amjmed.2011.12.013 22748400

[10] Lee HY Lee KS. Withdrawal of antihypertensive medication in young to middle-aged adults: a prospective, single-group, intervention study. Clin Hypertens. (2023) 29:1. 10.1186/s40885-022-00225-2 36593518 PMC9806446

[11] Kim S Shin DW Yun JM Hwang Y Park SK Ko YJ. Medication adherence and the risk of cardiovascular mortality and hospitalization among patients with newly prescribed antihypertensive medications. Hypertension. (2016) 67:506–12. 10.1161/HYPERTENSIONAHA.115.06731 26865198

[12] Carey RM Muntner P Bosworth HB Whelton PK. Prevention and control of hypertension: JACC health promotion series. J Am Coll Cardiol. (2018) 72:1278–93. 10.1016/j.jacc.2018.07.008 30190007 PMC6481176

[13] Böhm M Schumacher H Teo KK Lonn EM Mahfoud F Mann JFE. Achieved blood pressure and cardiovascular outcomes in high-risk patients: results from ONTARGET and TRANSCEND trials. Lancet. (2017) 389:2226–37. 10.1016/S0140-6736(17)30754-7 28390695

[14] Shin JH Jung MH Kwon CH Lee CJ Kim DH Kim HL. Disparities in mortality and cardiovascular events by income and blood pressure levels among patients with hypertension in South Korea. J Am Heart Assoc. (2021) 10:e018446. 10.1161/JAHA.120.018446 33719521 PMC8174369

[15] Seong SC Kim YY Park SK Khang YH Kim HC Park JH. Cohort profile: the national health insurance service-national health screening cohort (NHIS-HEALS) in Korea. BMJ Open. (2017) 7:e016640. 10.1136/bmjopen-2017-016640 28947447 PMC5623538

[16] Jung MH Lee SY Youn JC Chung WB Ihm SH Kang D. Antihypertensive medication adherence and cardiovascular outcomes in patients with cancer: a nationwide population-based cohort study. J Am Heart Assoc. (2023) 12:e029362. 10.1161/JAHA.123.029362 37421285 PMC10382088

[17] Mancia G Kreutz R Brunstrom M Burnier M Grassi G Januszewicz A. 2023 ESH guidelines for the management of arterial hypertension the task force for the management of arterial hypertension of the european society of hypertension: endorsed by the international society of hypertension (ISH) and the european renal association (ERA). J Hypertens. (2024) 42:194. 10.1097/HJH.0000000000003621 38033262

[18] Charlson ME Pompei P Ales KL MacKenzie CR. A new method of classifying prognostic comorbidity in longitudinal studies: development and validation. J Chronic Dis. (1987) 40:373–83. 10.1016/0021-9681(87)90171-8 3558716

[19] Choudhry NK Kronish IM Vongpatanasin W Ferdinand KC Pavlik VN Egan BM. Medication adherence and blood pressure control: a scientific statement from the American heart association. Hypertension. (2022) 79:e1–e14. 10.1161/HYP.0000000000000203 34615363 PMC11485247

[20] Mancia G Facchetti R Bombelli M Cuspidi C Grassi G. White-coat hypertension: pathophysiological and clinical aspects: excellence award for hypertension research 2020. Hypertension. (2021) 78:1677–88. 10.1161/HYPERTENSIONAHA.121.16489 34757765 PMC9634724

[21] McEvoy JW McCarthy CP Bruno RM Brouwers S Canavan MD Ceconi C. 2024 ESC guidelines for the management of elevated blood pressure and hypertension. Eur Heart J. (2024) 45:3912–4018. 10.1093/ehjcvp/pvae084 39210715

[22] Verdecchia P Angeli F Cavallini C Mazzotta G Garofoli M Martire P. The optimal blood pressure target for patients with coronary artery disease. Curr Cardiol Rep. (2010) 12:302–6. 10.1007/s11886-010-0112-y 20425161

[23] Glynn RJ. L'Italien GJ, Sesso HD, Jackson EA, Buring JE. Development of predictive models for long-term cardiovascular risk associated with systolic and diastolic blood pressure. Hypertension. (2002) 39:105–10. 10.1161/hy1201.097199 11799087

[24] Psaty BM Furberg CD Kuller LH Cushman M Savage PJ Levine D. Association between blood pressure level and the risk of myocardial infarction, stroke, and total mortality: the cardiovascular health study. Arch Intern Med. 2001:1183-92. 10.1001/archinte.161.9.1183 11343441

[25] Angeli F Reboldi G Verdecchia P. Hypertension and the J-curve phenomenon: implications for tight blood pressure control. Hypertens Res. (2013) 36:109–11. 10.1038/hr.2012.165 23154585

[26] Franklin SS Wong ND. Hypertension and cardiovascular disease: contributions of the framingham heart study. Glob Heart. (2013) 8:49–57. 10.1016/j.gheart.2012.12.004 25690263

[27] Tajeu GS Kent ST Huang L Bress AP Cuffee Y Halpern MT. Antihypertensive medication nonpersistence and low adherence for adults < 65 Years initiating treatment in 2007-2014. Hypertension. (2019) 74:35–46. 10.1161/HYPERTENSIONAHA.118.12495 31132956 PMC6914333

[28] Kronish IM Lynch AI Oparil S Whittle J Davis BR Simpson LM. The Association between antihypertensive medication nonadherence and visit-to-visit variability of blood pressure: findings from the antihypertensive and lipid-lowering treatment to prevent heart attack trial. Hypertension. (2016) 68:39–45. 10.1161/HYPERTENSIONAHA.115.06960 27217410 PMC4900942

[29] Derington CG Bress AP Herrick JS Jacobs JA Zheutlin AR Berchie RO. Antihypertensive medication regimens used by US adults with hypertension and the potential for fixed-dose combination products: the national health and nutrition examination surveys 2015 to 2020. J Am Heart Assoc. (2023) 12:e028573. 10.1161/JAHA.122.028573 37158068 PMC10381985

[30] Verma AA Khuu W Tadrous M Gomes T Mamdani MM. Fixed-dose combination antihypertensive medications, adherence, and clinical outcomes: a population-based retrospective cohort study. PLoS Med. (2018) 15:e1002584. 10.1371/journal.pmed.1002584 29889841 PMC5995349

[31] Bangalore S Kamalakkannan G Parkar S Messerli FH. Fixed-dose combinations improve medication compliance: a meta-analysis. Am J Med. (2007) 120:713–9. 10.1016/j.amjmed.2006.08.033 17679131

[32] Katz ME Mszar R Grimshaw AA Gunderson CG Onuma OK Lu Y. Digital health interventions for hypertension management in US populations experiencing health disparities: a systematic review and meta-analysis. JAMA Netw Open. (2024) 7:e2356070. 10.1001/jamanetworkopen.2023.56070 38353950 PMC10867699

[33] Mohan A Majd Z Johnson ML Essien EJ Barner J Serna O. A motivational interviewing intervention to improve adherence to aceis/arbs among nonadherent older adults with comorbid hypertension and diabetes. Drugs Aging. (2023) 40:377–90. 10.1007/s40266-023-01008-6 36847995 PMC9969383

[34] Hedegaard U Kjeldsen LJ Pottegard A Henriksen JE Lambrechtsen J Hangaard J. Improving medication adherence in patients with hypertension: a randomized trial. Am J Med. (2015) 128:1351–61. 10.1016/j.amjmed.2015.08.011 26302142

[35] Rosendo-Silva B Ortigosa-Ferreira AC Prazeres F Caramelo F Santiago LM Rosendo I. Systematic review of motivational interventions to improve adherence to medication in patients with hypertension and meta-analysis. Hipertens Riesgo Vasc. (2023) 40:174–96. 10.1016/j.hipert.2023.04.003 39492317

[36] Aremu TO Oluwole OE Adeyinka KO Schommer JC. Medication adherence and compliance: recipe for improving patient outcomes. Pharmacy. (2022) 10:106. 10.3390/pharmacy10050106 36136839 PMC9498383

