# Supplementary material for: Impact of blood pressure and medication adherence on clinical outcomes in patients with hypertension
Source: Front Med (Lausanne). 2025 Apr 28;12:1564791. doi: 10.3389/fmed.2025.1564791 (PMC12066674; doi:10.3389/fmed.2025.1564791)
Supplement: Supplementary file 2 [file Data_Sheet_1.docx]

**Supplementary Material**

Supplementary Figure S1. Risk of all-cause death stratified by systolic blood pressure and medication adherence

1. The forest plot of HRs for all-cause death according to SBP categories and medication adherence levels


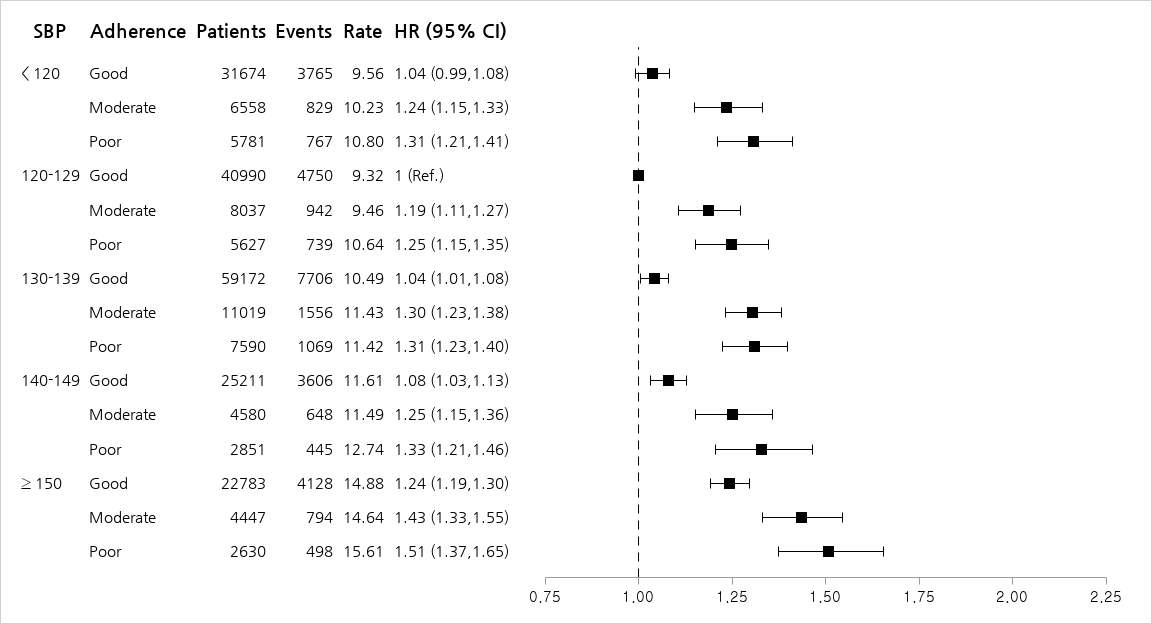


1. The forest plot of HRs for all-cause death according to medication adherence levels and SBP categories.


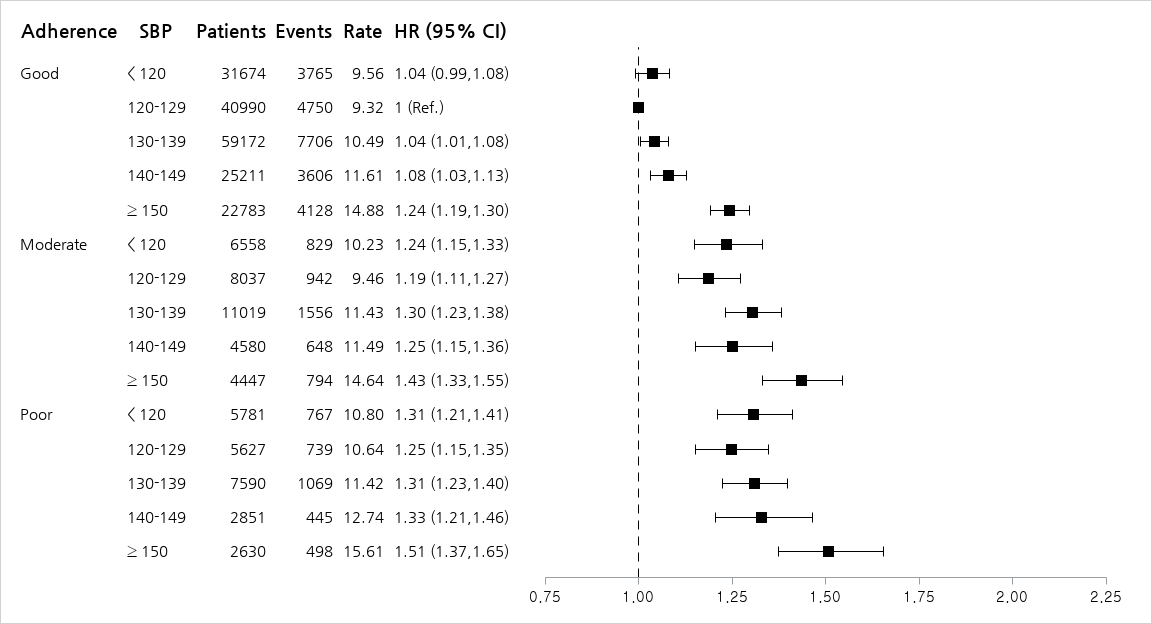


CI, confidence interval; HR, hazard ratio; SBP, systolic blood pressure

Supplementary Figure S2. Risk of hospitalization due to myocardial infarction stratified by systolic blood pressure and medication adherence

(A) The forest plot of HRs for hospitalization due to myocardial infarction according to SBP categories and medication adherence levels


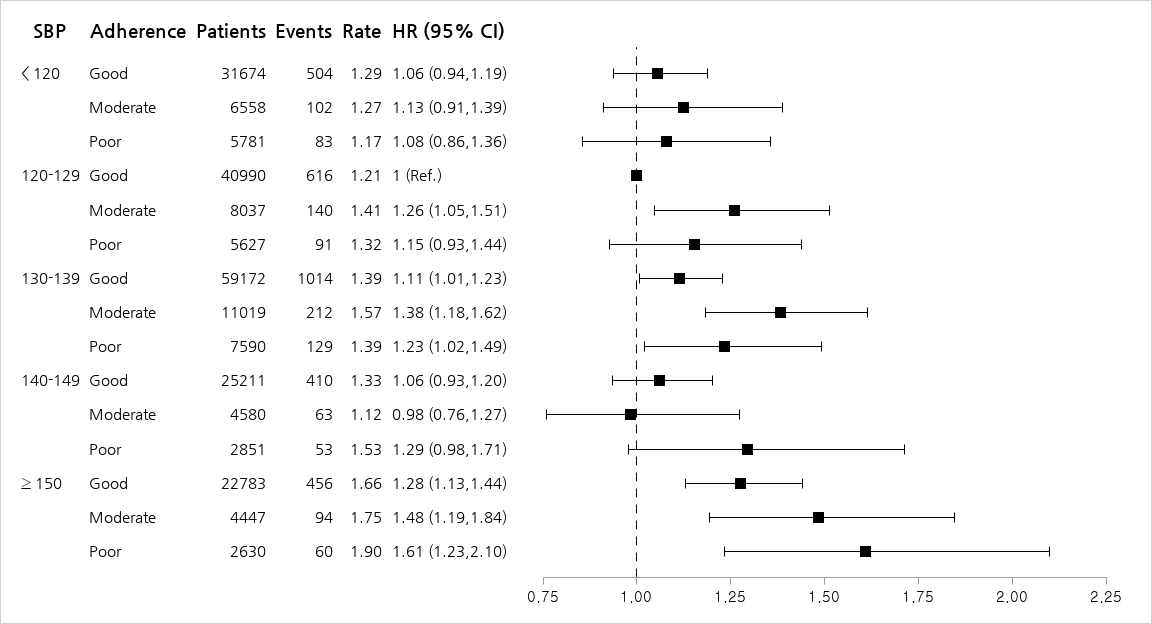


(B) The forest plot of HRs for hospitalization due to myocardial infarction according to medication adherence levels and SBP categories


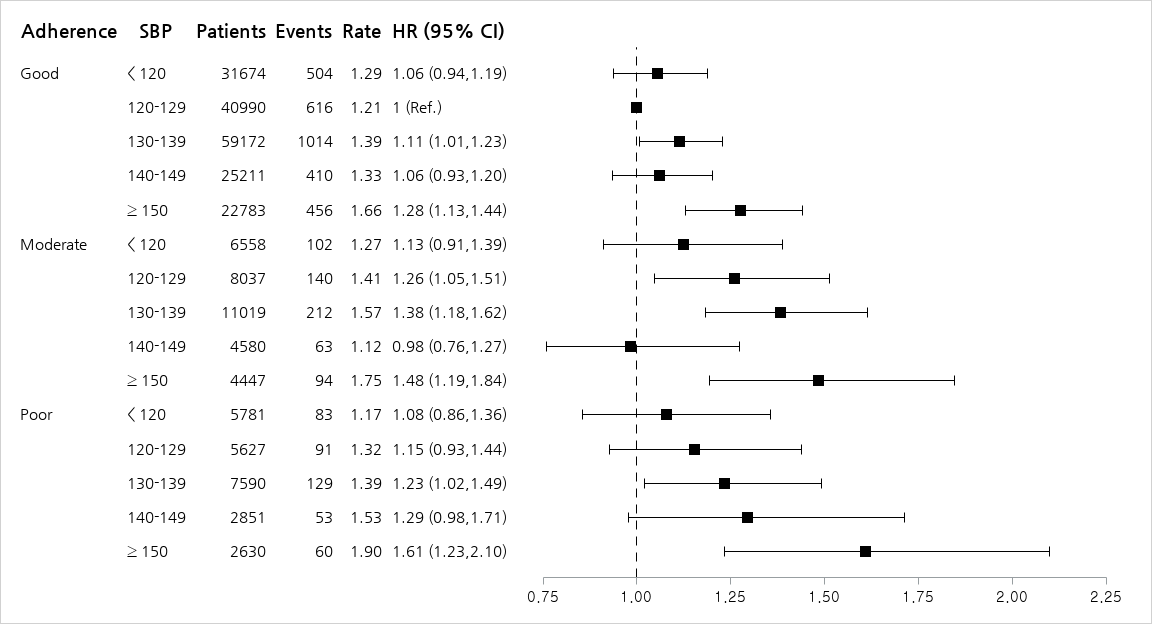


Supplementary Figure S3. Risk of ischemic stroke stratified by systolic blood pressure and medication adherence

1. The forest plot of HRs for ischemic stroke according to SBP categories and medication adherence levels


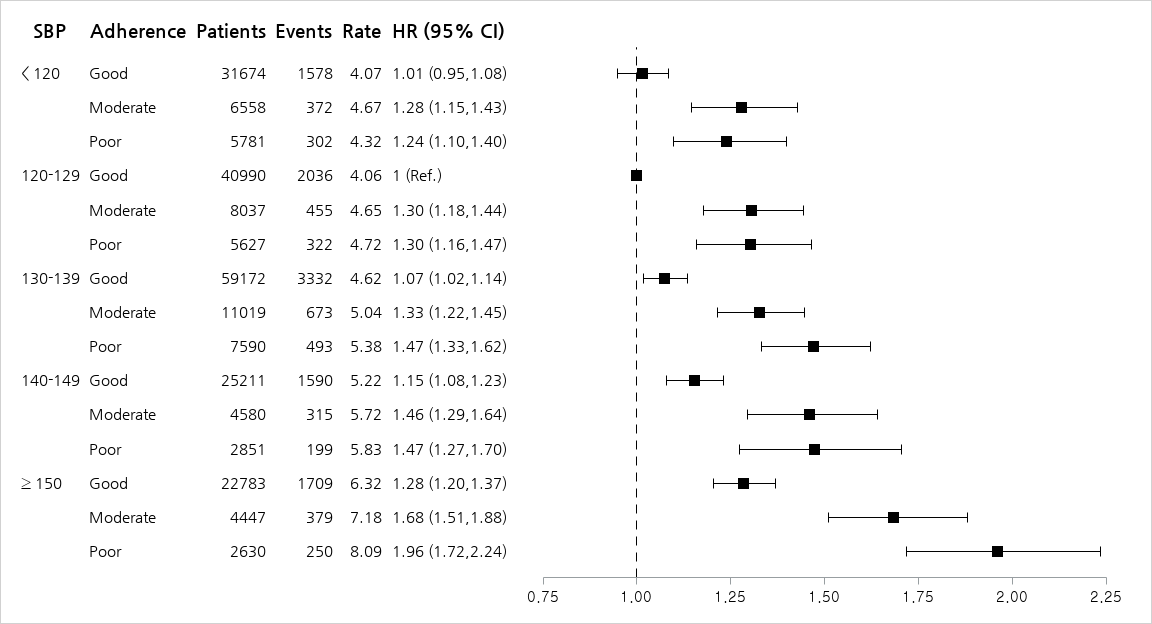


1. The forest plot of HRs for ischemic stroke according to medication adherence levels and SBP categories


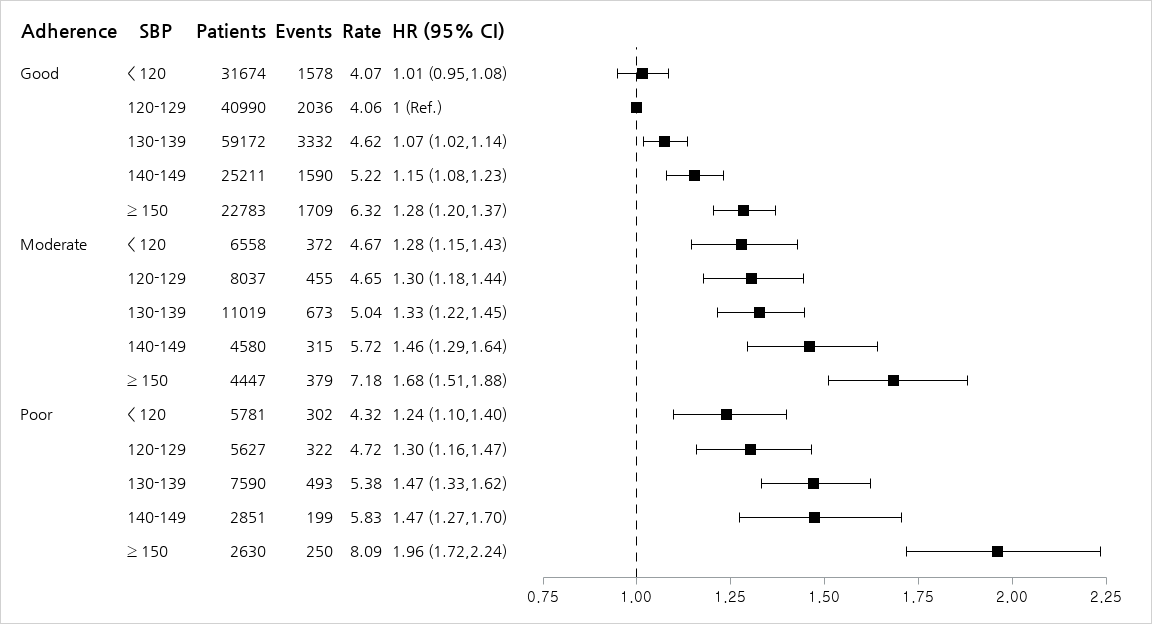


Supplementary Figure S4. Risk of hemorrhagic stroke stratified by systolic blood pressure and medication adherence

1. The forest plot of HRs for hemorrhagic stroke according to SBP categories and medication adherence levels


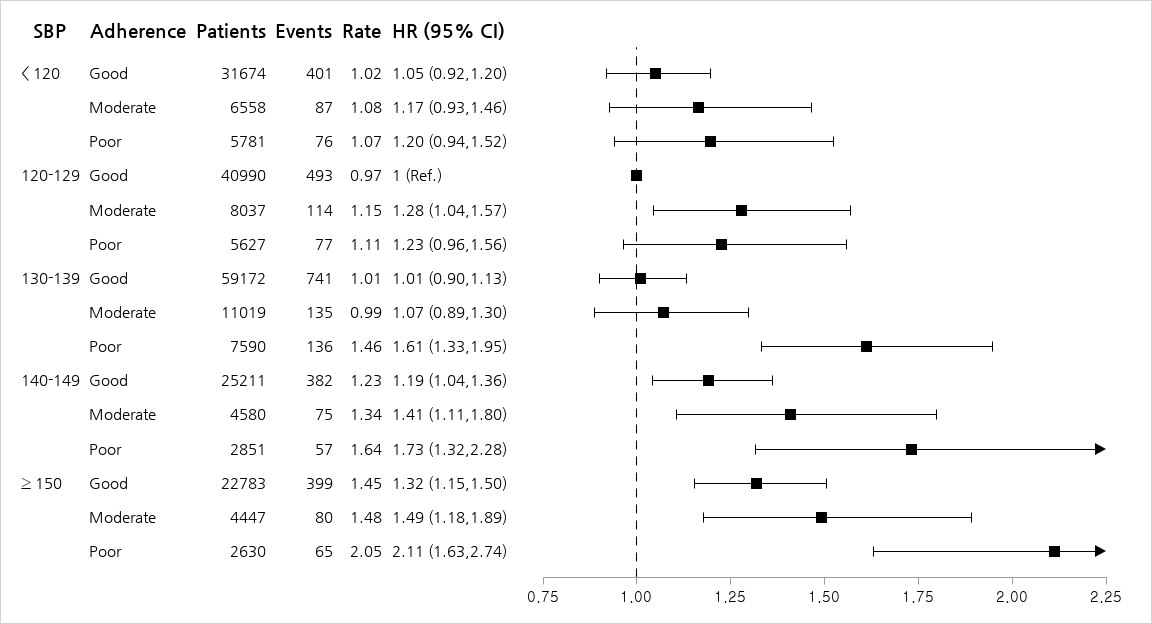


1. The forest plot of HRs for hemorrhagic stroke according to medication adherence levels and SBP categories


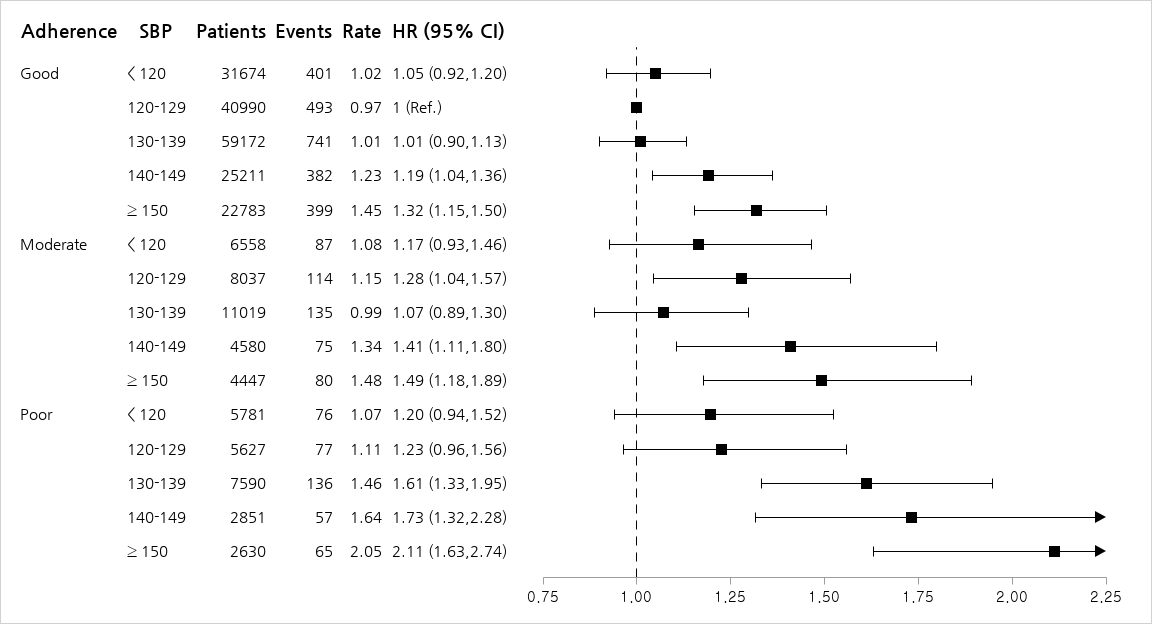


Supplementary Figure S5. Risk of heart failure stratified by systolic blood pressure and medication adherence

1. The forest plot of HRs for heart failure according to SBP categories and medication adherence levels


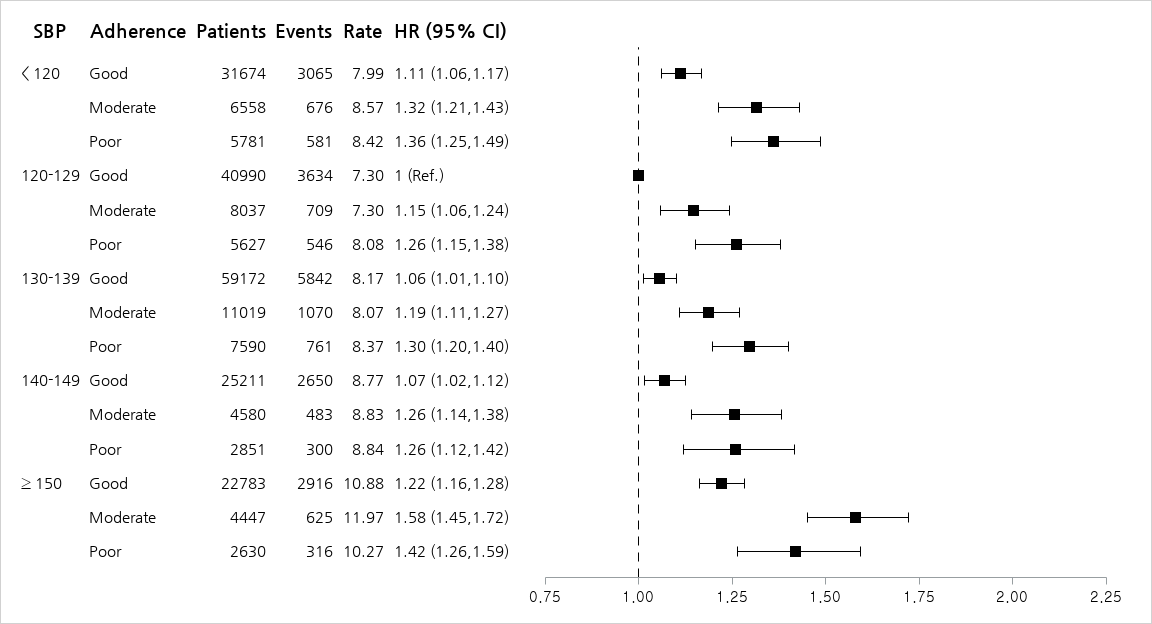


1. The forest plot of HRs for heart failure according to medication adherence levels and SBP categories


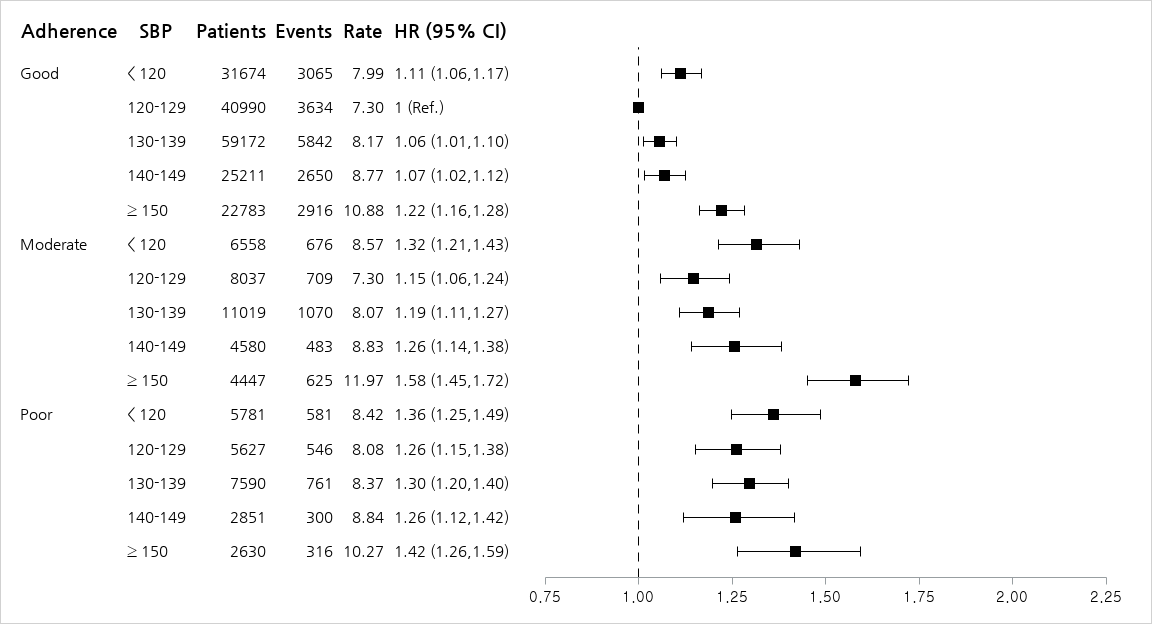


Supplementary Table S1. Risk of primary outcomes according to systolic blood pressure and medication adherence

|  |  |  |  | **Model 1** | | | **Model 2** | | | **Model3** | | |
| --- | --- | --- | --- | --- | --- | --- | --- | --- | --- | --- | --- | --- |
|  | **n** | **Events** | **Incidence Rate** | **HR** | **95% CI** | ***P*-value** | **HR** | **95% CI** | ***P-*value** | **HR** | **95% CI** | ***P*-value** |
|  |  |  | **per 1,000 PY** |  |  |  |  |  |  |  |  |  |
| **SBP (mmHg)** |  |  |  |  |  |  |  |  |  |  |  |  |
| < 110 | 9698 | 2325 | 20.48 | 1.235 | 1.181-1.291 | <.0001 | 1.205 | 1.152-1.26 | <.0001 | 1.178 | 1.127-1.232 | <.0001 |
| 110-119 | 34315 | 7523 | 18.46 | 1.05 | 1.02-1.081 | 0.0009 | 1.043 | 1.013-1.074 | 0.0045 | 1.034 | 1.005-1.064 | 0.0234 |
| 120–129 | 54654 | 11697 | 18.01 | 1 | (Ref.) |  | 1 | (Ref.) |  | 1 | (Ref.) |  |
| 130–139 | 77781 | 18453 | 20.10 | 1.051 | 1.027-1.076 | <.0001 | 1.05 | 1.026-1.075 | <.0001 | 1.056 | 1.031-1.08 | <.0001 |
| 140–149 | 32642 | 8203 | 21.49 | 1.058 | 1.029-1.089 | <.0001 | 1.059 | 1.03-1.09 | <.0001 | 1.068 | 1.038-1.099 | <.0001 |
| ≥ 150 | 29860 | 9147 | 26.86 | 1.231 | 1.198-1.265 | <.0001 | 1.218 | 1.185-1.252 | <.0001 | 1.238 | 1.204-1.272 | <.0001 |
| **Adherence** |  |  |  |  |  |  |  |  |  |  |  |  |
| Good | 179830 | 42636 | 20.11 | 1 | (Ref.) |  | 1 | (Ref.) |  | 1 | (Ref.) |  |
| Moderate | 34641 | 8523 | 21.02 | 1.196 | 1.168–1.224 | <0.0001 | 1.196 | 1.168–1.224 | <0.0001 | 1.201 | 1.173–1.229 | <0.0001 |
| Poor | 24479 | 6189 | 21.71 | 1.266 | 1.232–1.300 | <0.0001 | 1.269 | 1.235–1.303 | <0.0001 | 1.262 | 1.229–1.297 | <0.0001 |

CI, confidence interval; HR, hazard ratio; SBP, systolic blood pressure; PY, person-year

Model 1: adjusted for age and sex.

Model 2: Adjusted for age, sex, body mass index, smoking status, alcohol consumption, physical activity, household income, fasting glucose, and total cholesterol.

Model 3: adjusted for age, sex, body mass index, smoking status, alcohol consumption, physical activity, household income, Charlson comorbidity index, and use of glucose-and lipid-lowering drugs

Supplementary Table S2. Risk of primary outcomes stratified by medication adherence and systolic blood pressure

|  |  |  |  |  | Model 1 | | | Model 2 | | | Model 3 | | |
| --- | --- | --- | --- | --- | --- | --- | --- | --- | --- | --- | --- | --- | --- |
| **Adherence** | **SBP (mmHg)** | **n** | **Events** | **Incidence Rate** | **HR** | **95% CI** | ***P*-** | **HR** | **95% CI** | ***P*-** | **HR** | **95% CI** | ***P*-value** |
|  |  |  |  | **per 1,000 PY** |  |  | **value** |  |  | **value** |  |  |  |
| Good | < 110 | 6678 | 1589 | 20.25 | 1.245 | 1.18-1.313 | <.0001 | 1.217 | 1.154-1.284 | <.0001 | 1.191 | 1.129-1.257 | <.0001 |
|  | 110–119 | 24996 | 5375 | 18.05 | 1.049 | 1.013-1.085 | 0.0064 | 1.041 | 1.006-1.077 | 0.0207 | 1.032 | 0.997-1.068 | 0.0697 |
|  | 120–129 | 40990 | 8635 | 17.68 | 1 (Ref.) | - |  | 1 (Ref.) | - |  | 1 (Ref.) | - |  |
|  | 130–139 | 59172 | 13888 | 19.85 | 1.056 | 1.028-1.085 | <.0001 | 1.055 | 1.027-1.083 | 0.0001 | 1.059 | 1.031-1.088 | <.0001 |
|  | 140–149 | 25211 | 6268 | 21.22 | 1.065 | 1.031-1.1 | 0.0002 | 1.064 | 1.03-1.099 | 0.0002 | 1.07 | 1.036-1.105 | <.0001 |
|  | ≥ 150 | 22783 | 6881 | 26.43 | 1.225 | 1.187-1.264 | <.0001 | 1.211 | 1.173-1.25 | <.0001 | 1.227 | 1.189-1.267 | <.0001 |
| Moderate | < 110 | 1461 | 350 | 20.56 | 1.12 | 0.998-1.256 | 0.0532 | 1.1 | 0.981-1.234 | 0.1033 | 1.082 | 0.964-1.214 | 0.1801 |
|  | 110–119 | 5097 | 1168 | 19.41 | 1.059 | 0.983-1.14 | 0.1319 | 1.053 | 0.978-1.134 | 0.1701 | 1.049 | 0.974-1.13 | 0.207 |
|  | 120–129 | 8037 | 1737 | 18.28 | 1 (Ref.) | - |  | 1 (Ref.) | - |  | 1 (Ref.) | - |  |
|  | 130–139 | 11019 | 2691 | 20.80 | 1.06 | 0.998-1.126 | 0.0565 | 1.061 | 0.999-1.127 | 0.0544 | 1.067 | 1.004-1.134 | 0.0354 |
|  | 140–149 | 4580 | 1163 | 21.84 | 1.07 | 0.993-1.153 | 0.0743 | 1.075 | 0.998-1.158 | 0.0579 | 1.09 | 1.011-1.174 | 0.0239 |
|  | ≥ 150 | 4447 | 1414 | 27.99 | 1.283 | 1.196-1.377 | <.0001 | 1.27 | 1.183-1.363 | <.0001 | 1.292 | 1.204-1.387 | <.0001 |
| Poor | < 110 | 1559 | 386 | 21.39 | 1.203 | 1.074-1.348 | 0.0014 | 1.162 | 1.037-1.302 | 0.0096 | 1.135 | 1.013-1.271 | 0.0296 |
|  | 110–119 | 4222 | 980 | 19.74 | 1.012 | 0.932-1.099 | 0.7737 | 1.008 | 0.928-1.095 | 0.8517 | 0.997 | 0.918-1.083 | 0.9436 |
|  | 120–129 | 5627 | 1325 | 20.08 | 1 (Ref.) | - |  | 1 (Ref.) | - |  | 1 (Ref.) | - |  |
|  | 130–139 | 7590 | 1874 | 21.13 | 1.027 | 0.957-1.102 | 0.4623 | 1.024 | 0.954-1.098 | 0.5171 | 1.036 | 0.965-1.111 | 0.3297 |
|  | 140–149 | 2851 | 772 | 23.37 | 1.038 | 0.95-1.134 | 0.4143 | 1.035 | 0.947-1.132 | 0.4435 | 1.058 | 0.968-1.157 | 0.2137 |
|  | ≥ 150 | 2630 | 852 | 28.76 | 1.238 | 1.136-1.35 | <.0001 | 1.219 | 1.118-1.33 | <.0001 | 1.261 | 1.156-1.376 | <.0001 |

CI, confidence interval; HR, hazard ratio; SBP, systolic blood pressure; PY, person-year

Model 1: adjusted for age and sex.

Model 2: Adjusted for age, sex, body mass index, smoking status, alcohol consumption, physical activity, household income, fasting glucose, and total cholesterol.

Model 3: adjusted for age, sex, body mass index, smoking status, alcohol consumption, physical activity, household income, Charlson comorbidity index, and use of glucose-and lipid-lowering drugs
